# Exploring suicide ideation in university students: sleep quality, social media, self-esteem, and barriers to seeking psychological help

**DOI:** 10.3389/fpsyt.2024.1352889

**Published:** 2024-04-05

**Authors:** Miguel Landa-Blanco, Karol Romero, Ivin Caballero, Ernesto Gálvez-Pineda, María José Fúnes-Henríquez, Rina Romero

**Affiliations:** School of Psychological Sciences, National Autonomous University of Honduras, Tegucigalpa, Honduras

**Keywords:** suicide, sleep, social media, self-esteem, psychological help

## Abstract

The purpose of the current study is to analyze how variations in suicidal ideation scores can relate to sleep quality, social media consumption, self-esteem, and perceived barriers to seeking psychological help in a sample of university students in Honduras. A quantitative cross-sectional design was used. Self-reported data was collected from a non-random sample of 910 university students in Honduras; their average age was 24.03 years (*SD*=6.05). Most respondents were women (67%) with men accounting for 33% of the sample. Measurements included item 9 of the Patient Health Questionnaire-9, the Single-Item Sleep Quality Scale, Rosenberg’s Self-Esteem Scale, Barriers to Seeking Psychological Help Scale for College Students, and a self-reported questionnaire on social media. In response to the query, “Over the past two weeks, how frequently have you experienced thoughts that you would be better off dead or of hurting yourself?” 54% (*n*=495) of participants indicated “not at all” 18% (*n*=168) reported “several days” 14% (*n*=129) responded “more than half of the days” and 13% (*n*=118) stated “nearly every day”. The results from the ordinal logistic regression model indicate that sleep quality and self-esteem serve as protective factors associated with decreased suicide ideation. At the same time, a higher number of social media platforms used per week and perceived barriers to seeking psychological help increase suicide ideation. Altogether, these variables explained 19% of the variance in suicidal ideation scores. Suicidal ideation is highly prevalent among the sampled university students.

## 1 Introduction

Suicide remains a global public health concern, and its impact on university students is particularly alarming (1). The transition from adolescence to adulthood, coupled with the academic, social, and personal pressures associated with university life, places students at an elevated risk of experiencing mental health challenges (2), including suicidal thoughts (3). Suicidal ideation refers to contemplating self-harm or ending one’s life, ranging from fleeting thoughts to detailed plans (4).

While extensive research has addressed suicide among university populations, the focus on specific cultural settings, such as Honduras, is limited (5). Countrywide data from 2022 indicates a suicide rate of 6.6 per 100,000 inhabitants, averaging 47 suicides monthly. Most victims fall within the age range of 15 to 24 years old (6). Although many factors influence suicidal ideation, the current study will focus on specific hypothetical predictors, including sleep quality (7), self-esteem (8), social media consumption (9), and perceived barriers to seeking psychological help (10).

For instance, sleep is a fundamental biological need, essential for physical and mental wellbeing. Beyond simply recharging our bodies, sleep plays a critical role in regulating emotions, processing information, and consolidating memories (11). A growing body of research highlights the intricate link between sleep and mental health (12). A recent study suggests that the relative influence of self-reported sleep quality is more strongly linked to mental and physical well-being than chronotype and sleep duration (13). Sleep disturbances serve as both a symptom and a risk factor for various mental health issues, including suicidal ideation (14). Previous meta-analysis revealed consistent positive associations between different forms of sleep disturbance (overall, insomnia, hypersomnia, and nightmares) and suicidal thoughts and behaviors (7).

Similarly, the role of self-esteem in mental health is pivotal, as it influences an individual’s perception of their worth and competence across various domains (15). Self-esteem is a psychological concept involving an individual’s subjective assessment of competence, worth, and significance across social, cognitive, and emotional aspects (16, 17). Previous research suggests that self-esteem may serve as a protective factor for suicidal behaviors (18). Individuals with higher self-esteem tend to exhibit greater resilience, problem-solving skills, and social support (19–21), making them less prone to experiencing thoughts of self-harm during challenging circumstances. Conversely, low self-esteem may be related to feelings of worthlessness, social isolation, and cognitive distortions (22–25).

In a similar vein, exploring the intricate dynamics of social media consumption and its impact on mental health sheds light on another aspect of suicidal risk. The relationship between social media consumption and suicidal risk is complex and multifaceted. A recent study concluded that young individuals at risk of suicide who use the Internet for suicide-related reasons are more likely to have high levels of social anxiety. They primarily use the Internet to connect with others and find information (26). Factors such as social comparison (27), cyberbullying (28), and screen time (29) may contribute to an environment that may heighten the vulnerability of individuals to suicidal thoughts and behaviors. Moreover, increased exposure to negative messages promoting self-harm, along with the emulation of self-injurious behaviors observed online and the adoption of self-harming practices from shared videos, further amplify these risks. Notably, prolonged and problematic engagement on these platforms is linked to higher levels of distress, unmet mental health support needs, diminished self-rated mental health, and an increased likelihood of experiencing suicidal risk (30), including attempted suicide (9).

The complex interplay of factors contributing to suicide risk among university students underscores the necessity of targeted interventions and support mechanisms. An eight-country survey of college students concluded that only about a quarter of respondents expressed certainty in seeking treatment for future emotional issues. Among those hesitant to seek help, the most common reasons were the desire to handle the problem independently and the inclination to confide in friends or family. The preference to manage issues alone and feelings of embarrassment correlated with a decreased likelihood of seeking help (31).

It is worth noting that psychotherapy interventions are significantly effective in decreasing suicide intention (32). Specific profiles of students with elevated suicide risk, such as those with high internalizing and externalizing disorders and low social connectedness, may perceive more barriers to seeking psychological help (33). Therefore, resistance and perceived barriers to seeking psychological help may constitute a risk factor for suicide risk.

Identifying and addressing these barriers is essential for developing effective intervention strategies. Common barriers include stigma, fear of judgment, cultural differences, lack of awareness, mistrust, and concerns about confidentiality and the effectiveness of the treatment (10, 34, 35). By exploring these barriers, researchers and mental health professionals can tailor outreach and support efforts to address specific obstacles that may deter individuals from seeking help (36).

The purpose of the current study is to analyze how variations in suicidal ideation scores can relate to sleep quality, social media consumption, self-esteem, and perceived barriers to seeking psychological help in a sample of university students in Honduras. This research stems from a recognition of the limited existing literature on suicide ideation among university students in Honduras (5). While studies on mental health among this demographic are available (37), a comprehensive analysis articulating these key variables’ integration is notably absent. This study endeavors to bridge this gap by employing a multifaceted approach, which delves into the intricate interrelations among diverse factors contributing to suicidal ideation. An essential aspect of our research lies in incorporating attitudes toward seeking psychological help—a crucial yet frequently overlooked topic when investigating mental health-related factors (38–40).

## 2 Materials and methods

### 2.1 Participants

For the first trimester of 2023, The National Autonomous University of Honduras (UNAH), the country’s biggest tertiary university based on student census, had 44,227 students enrolled in its central campus. At a 99% confidence level and a 5% margin of error, the minimum required sample size was calculated at 654 participants. However, this number was overachieved, as 910 undergraduate students in the UNAH completed the survey.

The participants were recruited through non-random sampling, using institutional emails and in-person campus visits to all main faculties (>1,000 students). The instruments were administered online using Google Forms. Being 18 or older and being actively enrolled in the National Autonomous University of Honduras were considered inclusion criteria. The average age was 24.03 years (*SD*=6.05). Most respondents were women (67%; *n*=613), with men accounting for 33% (*n*=297) of the sample.

### 2.2 Instruments

#### 2.2.1 Patient health questionnaire-9

Suicide ideation was measured with the question, “over the last 2 weeks, how often have you been bothered by thoughts that you would be better off dead, or of hurting yourself?”, an item from the Patient Health Questionnaire- 9 (PHQ-9) (41). Responses for this item vary between 0 (“not at all”) to 3 (“nearly every day”), with higher scores indicating a higher frequency of suicidal ideation. This item of the PHQ-9 has been commonly used as a stand-alone measurement of suicidal ideation; there is evidence of its convergent validity with the widely used Mini-International Neuropsychiatric Interview suicidality module (42).

#### 2.2.2 Single-item sleep quality scale

Sleep quality was measured by the Single-Item Sleep Quality Scale (SQS) (43), which states, “During the past 7 days, how would you rate your sleep quality overall?”, with responses varying from 0 (terrible) to 10 (excellent). Previous research has concluded that the SQS has a strong convergent validity with the Pittsburgh Sleep Quality Index and the Morning Questionnaire-Insomnia (MQI), as well as known-group validity and adequate test-retest reliability (43).

#### 2.2.3 Rosenberg’s self-esteem scale

The Rosenberg’s Self-Esteem Scale was also included in the data collection process (44). This 10-item questionnaire is rated on a Likert-type response set varying between 1 (“strongly disagree”) to 4 (“strongly agree”), with total scores ranging from 10 (low self-esteem) to 40 (high self-esteem). In our sample, the Rosenberg’s Self-Esteem Scale achieved adequate reliability, *ω*=0.85, *CI* 95% [0.84; 0.86].

#### 2.2.4 Barriers to seeking psychological help scale for college students

Additionally, respondents completed the Barriers to Seeking Psychological Help Scale for College Students, a 17-item questionnaire that uses a Likert-type response set (1=“ strongly disagree”; 5=“ strongly agree”) (10). Total summative scores range from 17 (low perceived barriers) to 85 (high perceived barriers). This questionnaire achieved adequate internal consistency in our study, *ω*=0.93, *CI* 95% [0.92; 0.94].

#### 2.2.5 Social media consumption

Respondents were also presented with a multiple-choice list of 10 social media platforms, and they were asked to mark all the platforms they use at least once a week; the sum of these marks constitutes the score of social media consumption. This methodology has been previously used to study social media usage in students of the National Autonomous University of Honduras (45).

### 2.3 Data analyses

Given the measurement level of the outcome variable, an ordinal logistic regression model was used to assess unidirectional relationships between suicidal ideation (independent variable), self-reported sleep quality, social media consumption, self-esteem, and perceived barriers to seeking mental health services, which were assigned as hypothetical predictors. This analysis was complemented with bivariate Spearman’s correlation coefficients (*rho*). All hypotheses were tested at a 95% confidence level using Jamovi (46). All questions in the survey were required; therefore, no missing data was included in the study.

### 2.4 Ethical considerations

The Research Ethics Committee of the Faculty of Social Sciences of the National Autonomous University of Honduras approved this study under registration CEIFCS-2023-P2.

## 3 Results

In response to the query, “Over the past two weeks, how frequently have you experienced thoughts that you would be better off dead or of hurting yourself?” 54% (*n*=495) of participants indicated “not at all” 18% (*n*=168) reported “several days” 14% (*n*=129) responded “more than half of the days” and 13% (*n*=118) stated “nearly every day”. Interval analysis of this question yielded a mean score of 0.86 (*SD*=1.09). The description of all variables included in the study can be seen in **Table 1**.

**Table 1:** Descriptive statistics for the variables included in the study.

Descriptive statistics	Suicidal ideation	Barriers to Seeking Psychological Help	Sleep Quality	Self-esteem
Mean	0.86	41.32	5.29	28.43
Standard Deviation	1.09	14.83	2.63	6.32
Mininum	0	17	1	10
Maximum	3	85	10	40
Skewness	0.89	0.31	0.14	-0.30
Kurtosis	-0.66	-0.46	-0.91	-0.43
25-percentil	0	29	3	24
50-percentil	0	41	5	29
75-percentil	2	52	7	33

Higher mean scores indicate a higher variable intensity.

A bivariate correlation analysis indicates that suicidal ideation is positively and significantly associated with perceived barriers to seeking psychological help (*rho*=0.35, *p*<.001), while inversely related to self-esteem (*rho*=-0.49, *p*<.001) and sleep quality (*rho*=-0.23, *p*<.001); social media consumption is not correlated to suicidal ideation (*rho*=0.06, *p*=0.076). As a secondary analysis to our study, it is worth noting that barriers to seeking psychological help scores are inversely related to sleep quality (*rho*=-0.18, *p*<.001) and self-esteem (*rho*=-0.33, *p*<.001); there is also a positive correlation between self-esteem and sleep quality (*rho*=.30, *p*<.001). Social media consumption did not significantly correlate with any of the variables included in the study, see **Table 2**.

**Table 2:** Bivariate correlations between variables.

Variable	Statistic	Suicidal ideation	Sleep quality	Social media consumption	Barriers to seeking psychological help
Sleep quality	*rho*	-0.23***	—		
	*p-*value	< .001	—		
Self-esteem	*rho*	-0.49***	0.30***	0.02	-0.33***
	*p-*value	< .001	< .001	0.546	< .001
Social media consumption	*rho*	0.06	-0.01	—	
	*p-*value	0.076	0.661	—	
Barriers to seeking psychological help	*rho*	0.35***	-0.18***	0.01	—
	*p-*value	< .001	< .001	0.996	—

***p <.001.

The ordinal logistic regression model output indicates that sleep quality (*β*=-0.07, *p*=0.014) and self-esteem (*β*=-0.16, *p*<.001) serve as protective factors associated with decreased suicide ideation. At the same time, a higher amount of social media platforms used per week (*β*=0.10, *p*=0.043) and perceived barriers to seeking psychological help (*β*=0.03, *p*<.001) increase suicide ideation. Altogether, these variables explained 19% of the variance in suicidal ideation scores, see **Table 3**.

**Table 3:** Ordinal logistic regression model explaining variations in suicidal ideation scores.

Predictor	Estimate	*SE*	*Z*	*p*	*OR*	95% *CI OR*	
						*LL*	*UL*
Sleep quality	-0.07	0.03	-2.45	**0.014**	0.93	0.88	0.99
Social media consumption	0.1	0.05	2.02	**0.043**	1.10	1.00	1.21
Self-esteem	-0.16	0.01	-12.42	**< .001**	0.85	0.83	0.87
Barriers to seeking psychological help	0.03	0.01	6.57	**< .001**	1.03	1.02	1.04

SE, Standard Error; OR, Odds Ratio, CI, Confindence Interval; LL, Lower Limit; UL, Upper Limit. Overall model test: χ^2^(4)=321.72, p<.001, Nagelkerke’s r^2^=.19. Variance Inflation Factor (VIF) values are acceptable (VIF 1.02 – 1.12), indicating no multicollinearity. Based on Durbin-Watson (DW) statistic, there is no evidence of autocorrelation (DW=1.92, p=.230). Significant p-values (<.05) are presented in bold.

## 4 Discussion

Our study findings indicate that suicidal ideation is highly prevalent in the sampled university students. This outcome is significantly influenced by self-esteem and sleep quality, acting as protective factors, whereas social media consumption and perceived barriers to seeking psychological help emerge as risk factors. The practical implications of these findings for mental health systems are profound.

For instance, implementing programs or resources that enhance self-esteem and prioritize healthy sleep habits may contribute to reducing the incidence of suicidal thoughts among at-risk populations. In this sense, recent research suggests that mindfulness-based approaches may be helpful to improve self-esteem, sleep quality, and suicide risk (47–49).

On the other hand, addressing social media consumption and perceived barriers to seeking mental health services as risk factors emphasizes the need for targeted interventions, particularly in injury prevention, to mitigate the potential adverse effects of excessive social media usage on mental health. In this sense, reducing screen time may result in significant reductions in suicidal behaviors (50). Moreover, algorithms supervising social media content can potentially be used to detect and prevent suicide behaviors (51).

Additionally, reducing perceived barriers to seeking psychological services, such as stigma or accessibility issues, is crucial for creating a supportive environment that encourages individuals to seek help when needed (40). Key strategies for addressing stigma include increasing mental health awareness, fostering social contact, leveraging advocacy from influential figures or groups, and implementing anti-discriminatory legislation (52).

Universities should implement proactive measures to screen for suicide risk among students, focusing on early detection and support (53). Regular mental health assessments, conducted confidentially, can identify those at risk. Additionally, campuses must provide accessible mental health services, including counseling and crisis intervention. 24/7 hotlines, peer support programs, and educational resources should be readily available. Since many individuals at risk of suicide often prefer self-help resources (54), promoting such resources within the university community could be beneficial at a psychoeducational level. Creating a stigma-free environment is crucial to encourage students to seek professional psychological help without fear of judgment. By integrating comprehensive mental health initiatives, universities can foster a supportive community and address the complex challenges students may face.

Psychological interventions, such as Cognitive Behavioral Therapy (CBT) and Dialectical Behavioral Therapy (DBT), have demonstrated effectiveness in mitigating suicidality by addressing various triggers for self-harm and suicidal thoughts (55, 56). Approaches like brief CBT (57), web-based CBT (58), and DBT have shown promise in reducing suicidal ideation and preventing subsequent suicide attempts and hospital visits (59). Measures such as restricting access to lethal means have shown significant reductions in suicide rates (60). Follow-up and monitoring strategies, including interventions like Brief Intervention and Contact (BIC) and peer support (61, 62), have been effective in decreasing suicidal behavior and fostering ongoing engagement with treatment, highlighting the importance of sustained support beyond initial interventions.

Recognizing the limitations of our study, we note that the non-probabilistic sampling method hinders generalizability. Moreover, as the study encompassed a diverse array of university students, it did not undertake specific screening for various conditions that could enhance insight into the prevalence and contributing factors of suicidal risk. For example, no data was collected regarding respondents’ socioeconomic status and cultural background, which previous research indicates relates to the topic (63, 64). Subsequent research endeavors should account for a broader spectrum of criteria, encompassing students diagnosed with mental health disorders and substance abuse issues.

Furthermore, the instruments used in the study have not previously been validated in the Honduran population; future studies should focus on determining the psychometric properties of the questionnaires in this country. Additionally, validating specific instruments like the Columbia–Suicide Severity Rating Scale (65) and The Personal Suicide Stigma Questionnaire (PSSQ) (66) would be a valuable input to clinicians in Honduras.

Despite being brief and practical, another limitation is that the reliance on single-item measures for assessing suicidal ideation and sleep quality provides a limited perspective on these variables. Additionally, all data being cross-sectional and self-reported introduces potential bias due to the sensitive nature of the variables. Future research stemming from this study could enhance generalizability by using probabilistic sampling methods, developing more nuanced measures for variables like suicidal ideation and sleep quality, and investigating the feasibility of integrating objective data sources to address potential biases associated with self-reporting. Longitudinal studies could examine the identified variables’ temporal dynamics and causal relationships.

In conclusion, the results of our study suggest a high prevalence of suicidal ideation among the university students sampled. This occurrence is notably impacted by self-esteem and sleep quality, which serve as protective factors, while social media usage and perceived obstacles to seeking psychological assistance are identified as risk factors. Overall, we believe our research contributes valuable insights to injury prevention strategies, highlighting the need for targeted interventions and support systems to mitigate the risk of suicidal behavior within this specific demographic. Understanding these factors is crucial for developing evidence-based preventive measures and fostering a safer environment for university students.

## Data availability statement

The raw data supporting the conclusions of this article will be made available by the authors, without undue reservation.

## Ethics statement

The studies involving humans were approved by Comité de Ética en Investigación de la Facultad de Ciencias Sociales (UNAH). The studies were conducted in accordance with the local legislation and institutional requirements. The participants provided their written informed consent to participate in this study.

## Author contributions

ML-B: Conceptualization, Formal analysis, Investigation, Methodology, Project administration, Supervision, Writing – original draft, Writing – review & editing. KR: Conceptualization, Formal analysis, Investigation, Methodology, Writing – original draft. IC: Conceptualization, Formal analysis, Investigation, Methodology, Writing – original draft. EG-P: Conceptualization, Formal analysis, Investigation, Methodology, Writing – original draft. MF-H: Conceptualization, Formal analysis, Investigation, Methodology, Writing – original draft. RR: Conceptualization, Formal analysis, Investigation, Methodology, Writing – original draft.
